# Optimal Labeling Dose, Labeling Time, and Magnetic Resonance Imaging Detection Limits of Ultrasmall Superparamagnetic Iron-Oxide Nanoparticle Labeled Mesenchymal Stromal Cells

**DOI:** 10.1155/2013/353105

**Published:** 2013-03-19

**Authors:** Anders Bruun Mathiasen, Louise Hansen, Tina Friis, Carsten Thomsen, Kishore Bhakoo, Jens Kastrup

**Affiliations:** ^1^Cardiac Stem Cell Laboratory and Catheterization Laboratory, Rigshospitalet, Copenhagen University Hospital, Blegdamsvej 9, 2100 Copenhagen, Denmark; ^2^Department of Radiology, Rigshospitalet, Copenhagen University Hospital, Copenhagen, Denmark; ^3^Translational Molecular Imaging Group, Singapore Bioimaging Consortium, Agency for Science, Technology and Research (A∗-STAR), Singapore 138667

## Abstract

*Background.* Regenerative therapy is an emerging treatment modality. To determine migration and retention of implanted cells, it is crucial to develop noninvasive tracking methods. The aim was to determine *ex vivo* magnetic resonance imaging (MRI) detection limits of ultrasmall superparamagnetic iron-oxide (USPIO) labeled mesenchymal stromal cells (MSCs). *Materials and Methods.* 248 gel-phantoms were constructed and scanned on a 1.5T MRI-scanner. Phantoms contained human MSCs preincubated with USPIO nanoparticles for 2, 6, or 21 hours using 5 or 10 **μ**g USPIO/10^5^ MSCs. In addition, porcine hearts were scanned after injection of USPIO labeled MSCs. *Results.* Using 21 h incubation time and 10 **μ**g USPIO/10^5^ MSCs, labeled cells were clearly separated from unlabeled cells on MRI using 250.000 (*P* < 0.001), 500.000 (*P* = 0.007), and 1.000.000 MSCs (*P* = 0.008). At lower incubation times and doses, neither labeled nor unlabeled cells could be separated. In porcine hearts labeled, but not unlabeled, MSCs were identified on MRI. *Conclusions.* As few as 250.000 MSCs can be detected on MRI using 21 h incubation time and 10 **μ**g USPIO/10^5^ MSCs. At lower incubation times and doses, several million cells are needed for MRI detection. USPIO labeled cells can be visualized by MRI in porcine myocardial tissue.

## 1. Introduction

Stem cell therapy with potential to regenerate damaged myocardium is an emerging treatment modality for ischemic heart disease [1–3]. For future success of cardiac stem cell therapy, it is crucial to develop noninvasive tracking methods for determining the biodistribution and fate of the stem cells after delivery. 

Thus far, tracking of cardiovascular delivered stem cells in a clinical setting has been limited to direct cell labeling with radioisotopes and tracking with gamma-cameras, single-photon emission computed tomography, or positron emission tomography [4]. Although providing highly sensitive visualization, these methods are limited by low spatial resolution and short half-lives of radioisotopes from minutes to hours, thus only permitting short-term tracking of the cells. Other drawbacks are exposure to ionizing radiation and nontarget signal leakage.

Tracking of cells labeled with superparamagnetic iron-oxide (SPIO) or ultrasmall superparamagnetic iron-oxide (USPIO) nanoparticles using magnetic resonance imaging (MRI) offers high spatial resolution in combination with high soft tissue detail, without exposing the patient to ionizing radiation. Furthermore, the cells can be tracked for months. Cellular labeling methods with SPIO or USPIO are relatively simple, fast and inexpensive. 

Iron-oxide is nontoxic, since iron is a naturally occurring metal in the human body, and the iron oxide core is coated with biocompatible shell, allowing its eventual assimilation via endogenous metabolic iron cycles. The use of SPIO and USPIO labeling is clinically safe and does not influence cell function [5].

MRI tracking of SPIO and USPIO labeled cells has been utilized *in vivo* in rat, canine, and porcine models of myocardial infarction (MI) using a variety of delivery methods [6–13]. The labeled cells were tracked for up to 8 weeks after delivery. *In vivo* tracking of SPIO and USPIO labeled cells has not yet been utilized in a clinical cardiovascular setting, but both SPIO and USPIO have been used successfully in a number of noncardiovascular clinical studies [14–19]. 

There has been some concern that MRI signals from SPIO and USPIO labeled cells may originate from macrophages that have engulfed the labeled cells. This was seen in a few rat studies [6, 20], but the majority of animal studies have shown the opposite, that the MRI does in fact originate from the labeled cells and not macrophages [7–9, 11–13, 21, 22]. A general concern for cardiovascular cell therapy has been that the number of cells that remain in the heart after treatment may be limited to only a few percent. However, it has recently been demonstrated that these studies may be severely biased, as there is considerable spontaneous leaking of the radioisotopes used in these studies [23]. Therefore, the number of cells remaining in the heart after treatment may be as high as 60% one week after treatment.

For tracking of nonphagocytic cells, USPIO particles are probably more suitable than SPIO particles, due to higher cellular uptake [24] and longer plasmatic half-life [25]. The USPIO particles used in the present study (IODEX) have an additional cross-linking of the dextran coating compared to traditionally used SPIO and USPIO particles [26]. This stabilizes the iron core of the particles allowing for longer cell tracking periods. 

The aim of the present study was to determine *ex vivo* MRI detection limits of IODEX labeled human MSCs with respect to cell numbers and USPIO concentration and incubation period for future clinical application.

## 2. Materials and Methods

### 2.1. Isolation and Culture Expansion of MSCs

Bone marrow was obtained from the iliac crest by needle aspiration from healthy donors. The studies were conducted under local ethical approval. Mononuclear cells were then isolated by gradient centrifugation and cultured in complete medium consisting of Dulbecco's modified Eagle medium supplemented with HEPES and L-glutamine, (PAA Laboratories, Austria), 10% fetal bovine serum (PAA Laboratories, Austria), and 1% penicillin/streptomycin (Invitrogen, Austria). Cells were incubated at 37°C in humid air with 5% CO_2_. Medium was changed twice a week. The cells were grown to confluence before each passage. After two passages, the cells were washed with PBS (Invitrogen, Austria) and harvested with TrypLE Select (Invitrogen, Austria). Cells from each donor were characterized by flow cytometry for CD90, CD73, CD105, CD13, CD45, and CD34, in accordance with the minimal criteria for defining multipotent mesenchymal stromal cells [27]. 

### 2.2. USPIO Preparation

Tat-peptide derivatized USPIO nanoparticles coated with dextran (IODEX-TAT-FITC; 15–20 nm) were prepared in our laboratory using the method described by Josephson et al. [28]. Briefly, the dextran-coated USPIO nanoparticles were synthesized and subsequently conjugated with TAT-fluorescein isothiocyanate (FITC) peptide [GRKKRRQRRR GYK(FITC)C-NH2]. TAT-FITC was synthesized using FMOC-protected amino acid (2-(1-H-benzotriazol-2-yl)-1,1,3,3-tetramethyluronium hexafluorophosphate; HBTU) activation chemistry. The final iron concentration was 2.5 mg/mL, and the solution was sterilized by gamma-irradiation prior to use.

### 2.3. USPIO Labeling of MSCs

Dose titrating evaluation of iron concentrations added to cells and resulting amounts of iron bound to cells by Josephson et al. [28] revealed that a plateau phase was reached at 100 *μ*g iron per 10^6^ cells (10 *μ*g iron per 10^5^ cells). In the present study, we wanted to evaluate both this maximum dose of 10 *μ*g iron per 10^5^ cells and also the half of this dose, 5 *μ*g iron per 10^5^ cells, as this dose reached near optimum iron binding in the original titration study [28]. In the mentioned titrating study, cells were incubated overnight (18–21 hours), whereas animal studies using IODEX-TAT-FITC for labeling MSCs have used only 4–6 hours of incubation [13, 29]. In the present study, we evaluate the mentioned iron doses at 2, 6, and 21 hours of incubation.

MSCs were labeled by incubation with USPIO nanoparticles at a concentration of either 5 *μ*g iron per 10^5^ cells (half dose) or 10 *μ*g iron per 10^5^ cells (full dose) in complete medium for 2, 6, or 21 hours at 37°C in humid air with 5% CO_2_. Then, the cells were washed 3 times in PBS and harvested with TrypLE Select and centrifuged 5 min at 300 g. After centrifugation, the cells were resuspended in PBS, and the number of cells and cell viability was determined by propidium iodide staining using a NucleoCounter NC-100 (Chemometec, Denmark).

### 2.4. USPIO Iron Concentration

MSCs in a volume corresponding to 1 × 10^5^ cells were transferred to microfuge tubes and centrifuged for 5 min at 500 g. Cell pellet was frozen and stored at –20°C until date of quantification. Then, the cells were resuspended in 50 *μ*L PBS, hydrolysed for 30 min with 100 *μ*L 6 M HCl, and pH neutralized by addition of 60 *μ*L 10 M NaOH. The cells were centrifuged for 2 min at 1300 g, and 100 *μ*L supernatant was used for automatic iron quantitation by use of a Konelab 60i robot (Therma Electron, Finland). 

### 2.5. MRI-Phantoms

**Figure 1:**
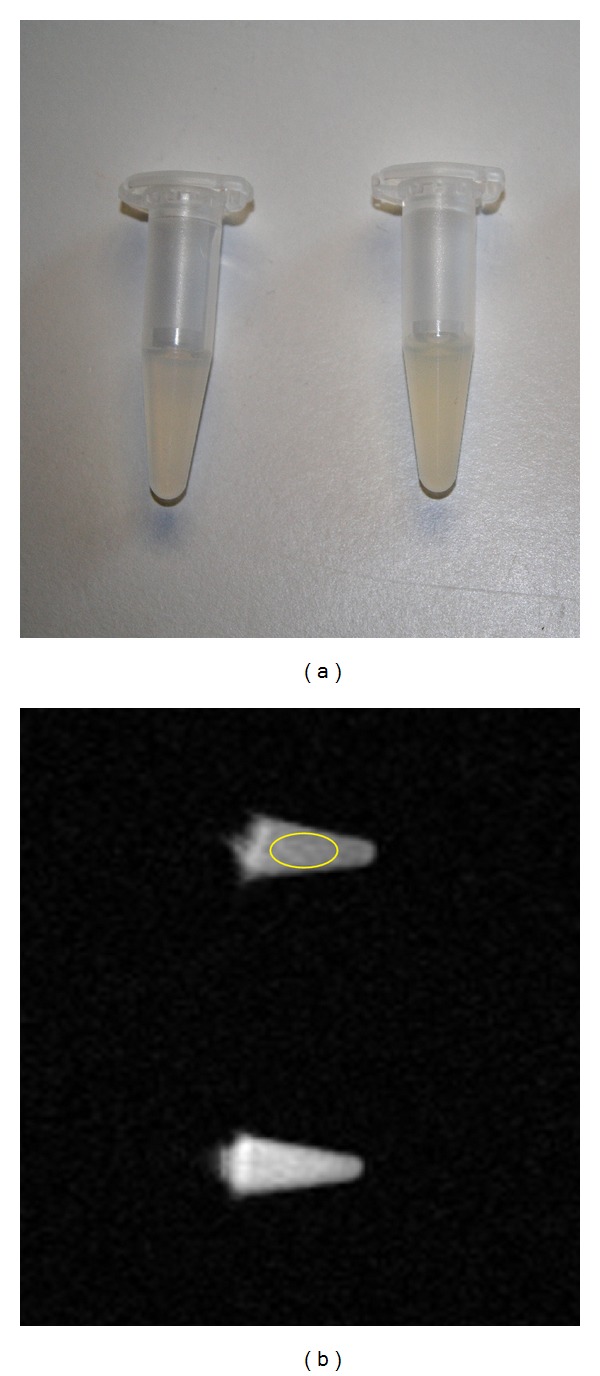
MRI phantoms. (a) Two phantoms containing USPIO labeled cells. (b) MRI image of 2 phantoms with an ellipsoid region of interest placed in the upper phantom. USPIO: ultrasmall superparamagnetic iron-oxide.

Labeled and unlabeled MSCs were transferred to microfuge tubes with 2.5 × 10^5^, 5 × 10^5^, or 1 × 10^6^ MSCs per tube. Tubes were centrifuged at 500 g for 5 minutes. The cells were then suspended in 500 *μ*L 1% agarose-gel. In total, 248 phantoms were constructed containing either unlabeled MSCs or MSCs labeled with half or full USPIO dose, incubated for 2, 6, or 21 hours. Two phantoms are shown in Figure 1(a). An overview of all MRI phantoms is provided in Table 1. A number of reference phantoms containing only agarose-gel were constructed as reference controls.

### 2.6. MRI Phantom Scanning Protocol and Image Analysis

Phantoms were scanned using a 1.5T GE Signa Excite HD MRI scanner with a 4-channel receive-transmit brain coil (GE Healthcare). Two phantoms and one reference phantom with no cells were scanned concurrently. Phantoms were placed in an Eppendorf tube rack, with the reference phantom in the center and a randomly selected MSC phantom on each side with 4 cm distance to the reference phantom. The rack was placed and fixated with tape on top of 4 other racks inside the coil to achieve a central position within the coil. Images were acquired using a brain-hemorrhage T2*-weighted gradient-echo (GRE) sequence with repetition-time (TR) = 620 ms, echo-time (TE) = 15.7 ms, flip-angle = 35°, matrix = 192 × 256, field of view (FOV) = 140 × 140 mm, and slice thickness = 7 mm.

Image analysis was performed using an Advantage Workstation AW4.3-05 (GE Healthcare). An ellipsoid region of interest (ROI) of 20 mm^2^ was placed on the images in the center of each phantom, avoiding the edges. The postprocessing tool produces mean intensity values for each ROI. Each pixel in the ROI is given an intensity value between 0 and 4095. The mean intensity value is the mean of these values for all the pixels in the ROI (Operators manual, GE Healthcare). For comparative analysis, the difference in mean intensity values between reference and cell phantom was used.  Figure 1(b) shows MRI image of 2 phantoms with an ellipsoid ROI placed in the upper phantom.

### 2.7. Porcine Hearts

Two hearts from freshly slaughtered pigs were placed and fixated with small wooden sticks in a polystyrene box. The hearts were MRI scanned before and after injection of MSCs. One heart was injected with 4 injections of USPIO labeled MSCs (full dose—21 hours incubation), each injection with approximately 2 × 10^6^ MSCs in 0.4 mL. The other heart received 4 injections with unlabeled cells. Care was taken that the hearts remained in the exact same position before and after injections.

### 2.8. MRI Scanning of Porcine Hearts

Hearts were scanned using a 1.5T Siemens Magnetom Avanto MRI scanner and a body matrix coil (Siemens AG, Germany). The scanning protocol was a thalassemia T2* weighted GRE sequence with TR = 200 ms, flip angle = 20°, matrix = 96 × 256, FOV = 135 × 180 mm, and slice thickness of 5 mm. The entire left ventricle was scanned with concurrent slice thickness of 5 mm with no gaps. The protocol produces 8 images for each slice, with different TE times (3.05, 5.89, 8.73, 11.57, 14.41, 17.25, 20.09, and 22.93 ms). 

### 2.9. Statistical Analysis

Statistical analysis was carried out using SPSS 20 (SPSS Inc., USA). One-way ANOVA tests were used for comparing cellular iron content and MRI intensity differences between groups. A *P* value < 0.05 was considered significant. If the ANOVA test of the groups was significant, a multiple group versus group comparison was made within the ANOVA procedure, to determine which of the groups differed. All *P* values in these tests were adjusted using the Bonferroni method to counteract the issue of multiple comparisons.

Normality was determined for each group with Kolmogorov-Smirnov and Shapiro-Wilk tests. Equal variances were determined with Levene's test for homogeneity of variances.

## 3. Results

### 3.1. Iron Content in MSCs

**Figure 2:**
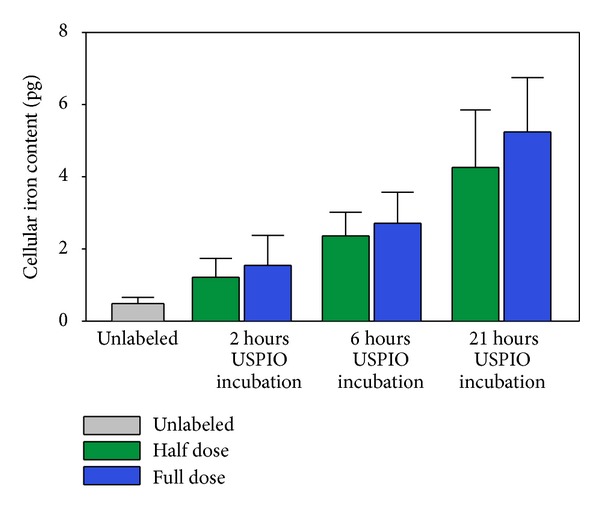
Cellular iron content. The iron content per cell was determined in unlabeled MSC and MSC incubated with half or full dose USPIO for 2, 6, and 21 hours. MSC: mesenchymal stromal cells. USPIO: ultrasmall superparamagnetic iron-oxide. USPIO dose—full: 10 *μ*g per 10^5^ cells; half: 5 *μ*g per 10^5^ cells.

Determination of the cellular iron load showed a positive correlation between iron content per cell and the length of the USPIO incubation period. The results are illustrated in Figure 2, and iron values and statistics are provided in Table 2. 

After 2 hours USPIO incubation time, the cellular iron content was only slightly higher than that of the unlabeled cells. This increase was only significant for the full USPIO dose compared to the unlabeled cells. After 6 hours USPIO incubation time, there was a highly significant increase in cellular iron content compared to unlabeled cells. When comparing to 2-hour incubation times, only the full USPIO dose was significantly higher after 6 hours. After 21 hours, the increase in cellular iron content was highly significant compared to both unlabeled and labeled cells for 2 and 6 hours at both USPIO doses. The cells labeled for 21 hours with the full USPIO dose also had significantly higher iron content than the cells labeled for 21 hours with only half USPIO dose.

### 3.2. MRI of USPIO Incubated Phantoms

**Figure 3:**
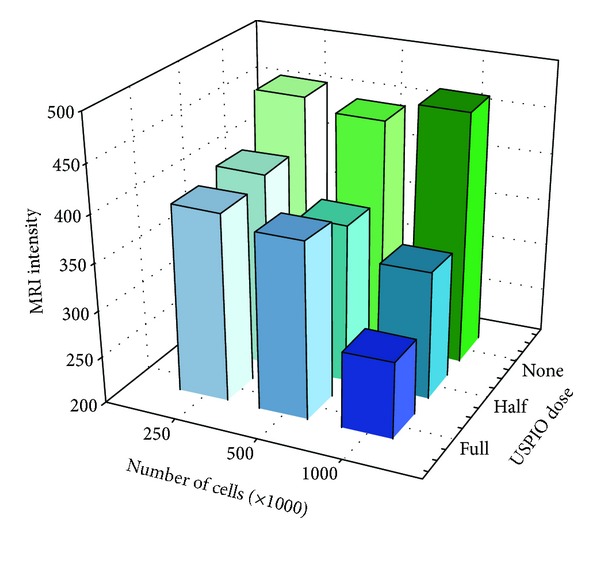
Absolute phantom MRI intensities after 21 hour USPIO incubation. MRI intensities are absolute mean values. USPIO: ultrasmall superparamagnetic iron-oxide. USPIO dose: full = 10 *μ*g per 10^5^ cells; half = 5 *μ*g per 10^5^ cells.

**Figure 4:**
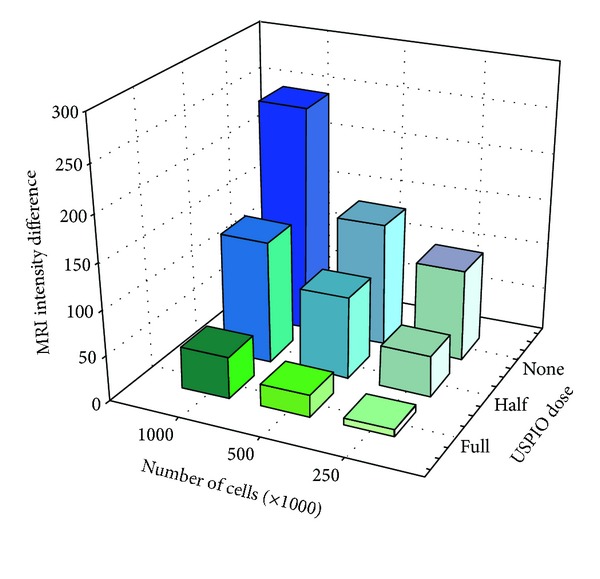
Phantoms intensity differences after 21-hour USPIO incubation. MRI intensity differences are the mean numeric difference between absolute MRI intensities of phantoms and reference gels. USPIO = ultrasmall superparamagnetic iron-oxide. USPIO dose: full = 10 *μ*g per 10^5^ cells; half = 5 *μ*g per 10^5^ cells.

Overall MRI intensity diminished with increasing cell numbers and USPIO dosage. A graphical illustration of the *absolute* MRI intensities of phantoms incubated with USPIO for 21 hours is provided in Figure 3, and an illustration of the numeric differences in MRI intensity compared to the reference gels is provided in Figure 4. The differences and statistics are shown in Table 3. 

USPIO labeled MSCs in amounts of 250.000, 500.000, and 1.000.000 could all be significantly separated on MRI from the same number of unlabeled cells, when using USPIO incubation time of 21 hours and full USPIO dosage. 

MSCs labeled with half USPIO dosage could not be separated from unlabeled MSCs at any concentration on MRI. With 2 and 6 hours of incubation time, it was not possible to differentiate between labeled and unlabeled cells at any dose or concentration on MRI (see Tables 4 and 5).

Therefore, the MRI detection limits are as low as 250.000 cells when using full USPIO dose and 21 hours of incubation time. For cells labeled with lower USPIO dose and lower incubation times, no significant difference was detected on MRI compared to unlabeled cells, and the detection limits for cells labeled using these conditions will therefore be at least several million cells. 

### 3.3. MRI of Porcine Hearts

**Figure 5:**
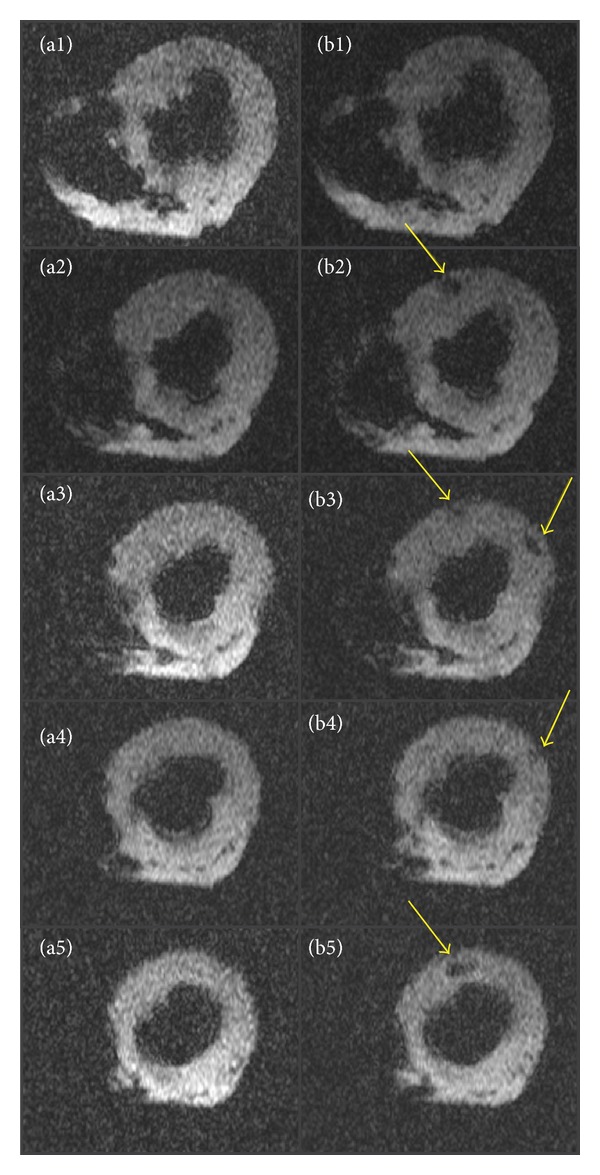
MRI images of porcine myocardium before and after USPIO labeled MSC injection. T2*-images of porcine myocardium before injection (a1–a5) and after injection (b1–b5) of USPIO-labeled MSCs. USPIO labeled MSCs are identified as hypointense areas (arrows). MSCs: mesenchymal stromal cells. USPIO: ultrasmall superparamagnetic iron-oxide.

There are distinct differences in the before and after images when looking at MRI images from porcine hearts receiving USPIO labeled MSCs (Figure 5). Hypointense areas can be identified in the after images which are equivalent to the USPIO labeled MSC injection areas. The figure images are with TE = 22.93 ms, which was the TE that gave the best visualization of the differences. 

**Figure 6:**
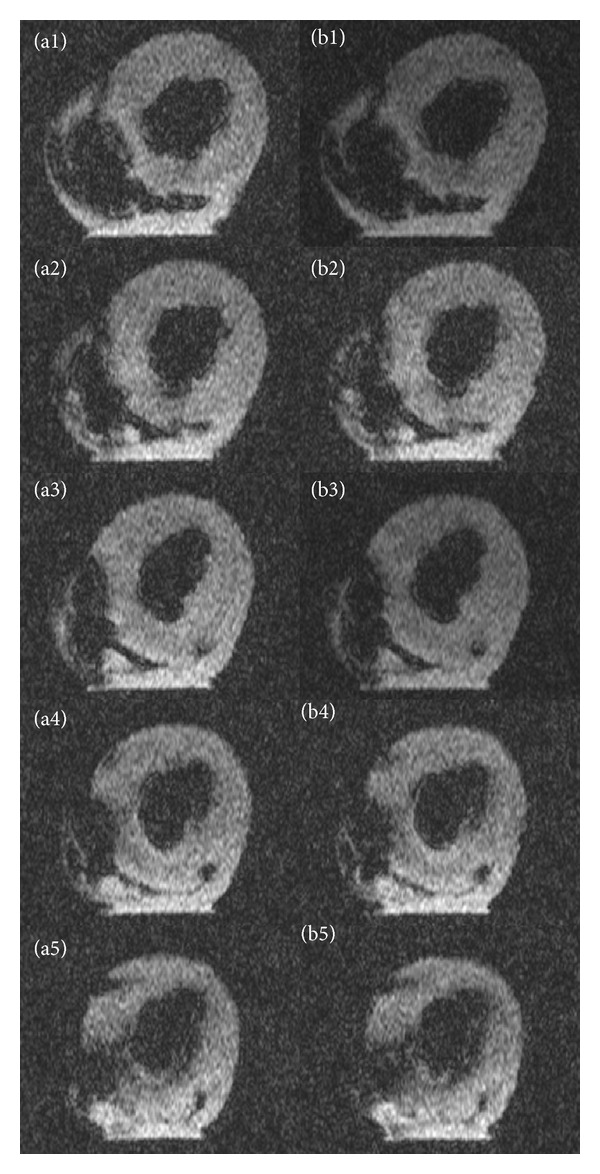
MRI images of porcine myocardium before and after unlabeled MSC injection. T2*-images of porcine myocardium before injection (a1–a5) and after injection (b1–b5) of unlabeled MSCs. Unlabeled cells cannot be identified. MSCs: mesenchymal stromal cells. USPIO: ultrasmall superparamagnetic iron-oxide.

MRI images from the heart receiving unlabeled cells were without visual differences; thus, unlabeled MSCs are undetectable on MRI (Figure 6). 

## 4. Discussion

In the present study, we have examined MRI detection limits of USPIO labeled MSCs *in vitro* with regard to cell numbers and USPIO incubation dosage and incubation time. The study demonstrated that an incubation period of 21 hours with USPIO is superior to 2 and 6 hours incubation times and that a USPIO incubation dose of 10 *μ*g per 10^5^ cells is preferable over 5 *μ*g per 10^5^ cells. In addition, USPIO labeled MSCs could be distinguished by MRI when injected into myocardial tissue. The hypointense MRI injection-regions were due to USPIO labeling, as unlabeled cells were not visible on MRI scans.

MRI tracking of cells labeled with iron-oxide based nanoparticles in cardiovascular disease has been utilized in a variety of animal studies. In one study, rats were subjected to MI and intramyocardial injection of SPIO labeled allogeneic MSCs [21]. The MSCs were injected in the border zone of the infarct area. Hypointense regions were visible on MRI in the entire 16-week followup period. In non-MI control rats injected with labeled cells, the hypointense regions were only visible on MRI for 1 week. This was also the case for MI control rats receiving SPIO particles alone. This indicated that cell retention is dependent on the presence of inflammation in the target tissue and also that SPIO particles from dead cells will be cleared from the area, and therefore the hypointense regions on MRI corresponded to SPIO particles within live cells. This was confirmed in histologic analysis done after 1, 16, and 20 weeks. SPIO-containing cells were identified at the injection site. Macrophage specific CD68 staining showed that macrophages were only present after 1 week and not after 16 and 20 weeks. The majority of CD68 positive cells did not contain iron, and most of the iron-containing cells did not express CD68. Thus, the originally labeled cells were present and not within macrophages. In a canine MI model, SPIO labeled MSCs were injected intramyocardially into the border zone of the infarct [7]. Injection sites were visible on MRI as hypointense regions for the entire 8 week followup period. Histology with Prussian Blue (PB) staining showed presence of SPIO containing cells well integrated within the tissue. 

Interesting results were found in a study using a rat MI model, where SPIO labeled MSCs were injected intramyocardially directly into the infarct lesion [6]. Hypointense regions were visible on MRI in the entire followup period of 4 weeks. In this study, postmortem tissue staining revealed that the delivery sites for both labeled and nonlabeled cells were infiltrated with inflammatory cells and that most MSCs did not survive. Similar results, where iron particles were engulfed in macrophages, were found in another rat study, where rats received intramyocardial injections of either xenogeneic human cells or allogeneic rat cells [20]. In both studies, the cells were injected directly into infarct lesion, whereas most other studies have injected their cells into the border zone of the infarct area. Perhaps the alternative injection site could explain the diverse results in these studies. 

Moreover, five other studies have also histologically evaluated intramyocardial injection [8] and intracoronary infusion of SPIO and USPIO labeled autologous MSCs [9, 12, 22] or endothelial progenitor cells [11] in porcine MI settings. In all these studies, hypointense regions were visible on MRI in the infarct region in the entire followup periods of 3–8 weeks. Interestingly and in strong contrast to the aforementioned rat study, post mortem analysis of sections of the infarct regions showed that SPIO and USPIO particles remained within the originally labeled cells and that these cells corresponded with hypointense MRI signals. Only one of the studies using SPIO labeling detected sparse amounts of macrophages in the tissue, and these were clearly separated from labeled cells [11]. These findings are supported by another study using a rat MI model, where USPIO labeled MSCs were intramyocardially injected. Histology in this study confirmed that the original labeled MSCs remained in the infarcted area up to 6 weeks after implantation using both MRI and histology [13]. 

A major concern with MRI tracking of SPIO and USPIO labeled cells has been that the obtained MRI signals could originate from macrophages that consumed the SPIO particles after cell death of the original labeled cells. The vast majority of animal studies have found the labeled cells to live at the injection sites with no signs of macrophages or other phagocytic cells. Therefore, concern for phagocytic engulfment of injected cells seems overrated and should not hinder future studies in this area.

Another concern has been that the number of cells that remain in the heart for a prolonged period of time may be limited. A number of animal studies have attempted to assess the number of cells that remain in the heart at different time points after intramyocardial injection. The study by Tran et al. [23] used ^111^In-oxine labeled culture expanded autologous MSCs from rats injected intramyocardially 4 weeks after MI. In an initial *in vitro* experiment, the spontaneous leaking rate of ^111^In-oxine from labeled MSCs was 28% per hour during the first 2 hours, and hereafter decreased rapidly. As a consequence of this, only 44% of ^111^In-oxine was retained within the MSCs at 2 hours, 27% at day 1 and 20% at day 7. Using a gamma-camera, ^111^In-oxine activity in the hearts after 2 hours was 27.1%, 17.4% at day 1, and 11.5% at day 7. Once these values were corrected for the ^111^In-oxine leakage measured at the same time-points, a mean constant value of 60% of injected MSCs could be estimated to be retained within the hearts over a period of 7 days. Similar studies in porcine using radioisotope labeled cells to determine long time cell retention have reported low long-term cell numbers similar to the uncorrected observations reported by Tran et al. [23]. These studies did not take into account the spontaneous radioisotope leakage from labeled cells, and long-term cell numbers would probably have been considerably higher if this had been taken into consideration [30, 31]. Long-term cell retention has also been evaluated in an allogeneic rat MI model, where female rats received intramyocardial injections with “male” MSCs [32]. Rats were sacrificed at different time intervals, and samples from the hearts were used to quantify male cell retention. At the initial time point, the cell retention varied from 9% to 80%, and after 6 weeks, the cell retention was down to 2% and 3.5%. However, the study has limitations as cell retention numbers were based on analysis of small myocardial samples that may not represent the whole myocardium. Moreover, the number of rats (only 2 to 5 in each group) was very small. That cells which may present in the heart for much longer were recently demonstrated using reporter genes in a mouse study where human CD34^+^ cells were detected up to 52 weeks after intramyocardial injection in the heart after MI [33].

MRI tracking of iron-oxide-based nanoparticles labeled cells has yet to be carried out in a clinical cardiovascular setting, but both SPIO and USPIO have been used successfully in different clinical studies. In one study, 10 patients with spinal cord injury received spinal injections of autologous CD34^+^ cells labeled with magnetic beads [14]. Treatment was safe and the labeled cells were tracked with MRI as hypointense signals in five patients up to 35 days after injection. In another study, SPIO labeled autologous dendritic cells were injected in lymph nodes in 11 melanoma patients [15]. The treatment was safe and labeled cells could be tracked on MRI. Histology of resected lymph nodes confirmed the presence of the original labeled cells. The SPIO labeled cells were negative for the macrophage marker CD68, indicating that the SPIO positive cells were not macrophages. SPIO labeled cells were also used in 15 patients with multiple sclerosis and 19 patients with amyotrophic lateral sclerosis [16]. Patients received spinal injections of SPIO labeled MSCs. Treatment was safe, and the labeled MSCs were visualized as hypointense signals with MRI. SPIO labeled pancreatic islets were transplanted into the livers of 4 patients with type 1 diabetes [18]. Treatment was safe, and labeled islets were identified as hypointense spots in 3 of 4 patients with MRI. In yet another study, one patient with brain trauma was transplanted with SPIO labeled neural cells [19]. Treatment was safe, and the labeled cells were tracked for 3 weeks. USPIO particles have been used with success in clinical settings as an MRI contrast in patients with stroke [17] and for sentinel node identification in cancer patients [34, 35].

For clinical use, commercial SPIO and USPIO products were available a few years ago, but these products have been taken of the market. The USPIO particles used in the present study (IODEX) were developed for clinical use and can be used as such, when produced under Good Manufacturing Practice (GMP) conditions. The IODEX particles are designed to remain stable for months without releasing the iron core. This is achieved with additional cross-linking of the dextran coating with epichlorohydrin [26]. In addition, the highly cationic HIV-tat peptide was used for internalization of intracellular MRI contrast agents [28]. This has a much higher cellular uptake than traditionally used such as poly-L-lysine or protamine sulfate.

As an intracellular contrast agent for nonphagocytic cells, we find that USPIO particles might be more suitable than SPIO particles for clinical use. In a comparison study, USPIO particles exhibited significantly higher uptake in nonphagocytic cells compared to SPIO particles [24]. These data suggest that smaller particles are internalized more efficiently into nonphagocytic cells. In contrast, another study found that uptake of SPIO was better than USPIO for nonphagocytic cells [36]. In this study, however, cells were only incubated for 4 hours, which may explain the lower USPIO uptake. As demonstrated in the present study, a longer incubation time is needed for optimal labeling with USPIO particles. For labeling of phagocytic cells though, SPIO particles might be more suitable, as SPIO particles are easily recognized and internalized into monocytes and macrophages [37]. Another advantage of USPIO particles is that they have longer plasmatic half-life (>36 hours) and this allows for longer cell tracking observation periods [25].

## 5. Conclusions

The present study demonstrated that to label MSCs for MRI tracking, the preferable USPIO incubation time and dosage were 21 hours and 10 *μ*g USPIO per 10^5^ MSCs, respectively. In porcine myocardial tissue, the USPIO labeled MSC could be visualized on MRI, whereas unlabeled cells could not. Clinical studies should be conducted, with MRI tracking of myocardial injected USPIO labeled MSCs in patients with heart disease to obtain important information on retention, migration, and efficacy of the cells after implantation.

## Figures and Tables

**Table 1:** Number of MRI phantoms.

Number of MSCs	USPIO dose	USPIO incubation time		
		2 hours	6 hours	21 hours
2.5 × 10^5^	full	13	10	13
5 × 10^5^	full	11	7	9
1 × 10^6^	full	12	7	8
2.5 × 10^5^	half	14	5	9
5 × 10^5^	half	11	5	8
1 × 10^6^	half	11	4	8
2.5 × 10^5^	0	14	5	12
5 × 10^5^	0	10	6	11
1 × 10^6^	0	12	5	8

MSC: mesenchymal stromal cell; USPIO: ultrasmall super-paramagnetic iron-oxide. USPIO dose—full = 10 *μ*g per 10^5^ cells; half = 5 *μ*g per 10^5^ cells.

**Table 2:** Cellular iron content.

Group	Iron content per cell	*n*	Multiple comparisons (Bonferroni corrected)						
			Group A	Group B	Group C	Group D	Group E	Group F	Group G
(A) Unlabeled	0.48 ± 0.17 pg	44	*— *	ns	*P* = 0.02	*P* < 0.001	*P* < 0.001	*P* < 0.001	*P* < 0.001
(B) Half dose, 2 hours incubation	1.22 ± 0.52 pg	17	*— *	*— *	ns	ns	*P* = 0.001	*P* < 0.001	*P* < 0.001
(C) Full dose, 2 hours incubation	1.54 ± 0.83 pg	17	*— *	*— *	*— *	ns	*P* = 0.03	*P* < 0.001	*P* < 0.001
(D) Half dose, 6 hours incubation	2.36 ± 0.65 pg	17	*— *	*— *	*— *	*— *	ns	*P* < 0.001	*P* < 0.001
(E) Full dose, 6 hours incubation	2.71 ± 0.86 pg	21	*— *	*— *	*— *	*— *	*— *	*P* < 0.001	*P* < 0.001
(F) Half dose, 21 hours incubation	4.26 ± 1.59 pg	37	*— *	*— *	*— *	*— *	*— *	*— *	*P* = 0.002
(G) Full dose, 21 hours incubation	5.24 ± 1.50 pg	44	*— *	*— *	*— *	*— *	*— *	*— *	*— *

Values are shown ± SD.

USPIO: ultrasmall super-paramagnetic iron-oxide. USPIO dose—full = 10 *μ*g per 10^5^ cells. half = 5 *μ*g per 10^5^ cells, ns: nonsignificant.

**Table 3:** MRI intensity differences after 21-hour USPIO incubation.

USPIO dose	1 × 10^6^ cells	5 × 10^5^ cells	2.5 × 10^5^ cells
Full	249 ± 102	135 ± 91	100 ± 64
Half	134 ± 82	91 ± 76	45 ± 92
Unlabeled	46 ± 58	25 ± 47	8 ± 53

Multiple comparisons	*P* < 0.001	*P* = 0.007	*P* = 0.008

Full versus unlabeled	*P* < 0.001	*P* = 0.006	*P* = 0.006
Full versus half	*P* = 0.034	ns	ns
Half versus unlabeled	ns	ns	ns

The MRI intensities are mean pixel intensities (values between 0 and 4095) of a 20 mm^2^ region of interest in the center area of each phantom, supplied by the imaging software. Values are shown ± SD.

USPIO: ultrasmall super-paramagnetic iron-oxide. USPIO dose—full = 10 *μ*g per 10^5^ cells. half = 5 *μ*g per 10^5^ cells, ns: non-significant.

**Table 4:** MRI intensity differences after 6-hour USPIO incubation.

USPIO dose	1 × 10^6^ cells	5 × 10^5^ cells	2.5 × 10^5^ cells
Full	74 ± 50	30 ± 26	38 ± 30
Half	57 ± 48	36 ± 32	19 ± 36
Unlabeled	47 ± 32	43 ± 20	6 ± 50
	ns	ns	ns

The MRI intensities are mean pixel intensities (values between 0 and 4095) of a 20 mm^2^ region of interest in the center area of each phantom, supplied by the imaging software. Values are shown ± SD.

USPIO: ultrasmall super-paramagnetic iron-oxide. USPIO dose—full = 10 *μ*g per 10^5^ cells. half = 5 *μ*g per 10^5^ cells. ns: non-significant.

**Table 5:** MRI intensity differences after 2-hour USPIO incubation.

USPIO dose	1 × 10^6^ cells	5 × 10^5^ cells	2.5 × 10^5^ cells
Full	59 ± 65	57 ± 39	60 ± 68
Half	39 ± 38	36 ± 62	41 ± 70
Unlabeled	71 ± 46	101 ± 41	55 ± 39
	ns	ns	ns

The MRI intensities are mean pixel intensities (values between 0 and 4095) of a 20 mm^2^ region of interest in the center area of each phantom, supplied by the imaging software. Values are shown ± SD.

USPIO: ultrasmall super-paramagnetic iron-oxide. USPIO dose—full = 10 *μ*g per 10^5^ cells; half = 5 *μ*g per 10^5^ cells. ns: non-significant.
